# Estrogen Receptor Signaling in Cancer

**DOI:** 10.3390/cancers12102744

**Published:** 2020-09-24

**Authors:** Simon P. Langdon

**Affiliations:** Cancer Research UK Edinburgh Centre and Edinburgh Pathology, MRC Institute of Genetics and Molecular Medicine, University of Edinburgh, Crewe Road South, Edinburgh EH4 2XU, UK; simon.langdon@ed.ac.uk

**Keywords:** estrogen receptor, breast cancer, ovarian cancer, prostate cancer, acute myeloid leukemia, imaging

## Abstract

Estrogen receptor signaling plays an important role not only in breast cancer but also in other cancers including ovarian cancer, prostate cancer and acute myeloid leukemia. This series of articles includes new findings of estrogen receptor co-regulators in breast and ovarian cancer with potential as novel targets for therapy. The major isoforms of estrogen receptor—namely estrogen receptor alpha and estrogen receptor beta—can have opposing functionality and these differing roles are described in reviews of estrogen signaling in prostate and ovarian cancers and acute myeloid leukemia. In breast cancer, mutated forms of the estrogen receptor can be selected for on endocrine treatment and frequently lead to treatment resistance while novel imaging techniques are being evaluated to monitor diagnosis and response to treatment. These developing research fields are overviewed.

The series of seven articles (two original articles and five reviews) in this Special Issue address estrogen receptor (ER) signaling pathways in cancer and is presented by international experts engaged in research across a variety of cancer types. While estrogen signaling has long been known to have a major role in breast cancer, there is emerging evidence that it can regulate function in other cancer types also. These include ovarian cancer, prostate cancers and acute myeloid leukemia (AML) and these are reviewed here alongside studies of breast cancer.

The two original articles both describe studies with ER-coregulators, which control the transcriptional activity of ER [1,2]. A study by Dominic Jones et al. [1] focusses on the role of the histone demethylases KDM3A and KDM4B in breast cancer and this report describes a co-operative mechanism regulating the chromatin transactivation of the ER. KDM3A and KDM4B regulate ER activity jointly via an autoregulatory loop that enhances the recruitment of each co-activating enzyme to chromatin. KDM3A primes chromatin for FOX1A binding and the recruitment of the ER transcriptional complex. Knockdown of both KDM3A and KDM4B produces a greater effect on ER activity and cell growth than the depletion of either enzyme alone [1]. As such, their targeting could be a possible therapeutic strategy. The study by Salvati et al. [2] investigated the role of the histone methyltransferase disruptor of telomeric silencing-1-like (DOT1L) in high grade serous ovarian cancer (HGSOC), the predominant form of epithelial ovarian cancer. Having demonstrated that HGSOC cancers with high co-expression of both estrogen receptor alpha (ERα) and DOT1L are associated with poor survival, the investigators used cell line models to investigate functional co-operation between ERα and DOT1L. The inhibition of DOT1L resulted in growth inhibition and modified the transcription of genes associated with invasion/migration and cell signaling in addition to the transcription of *ESR1* (the ERα gene). The inhibition of both DOT1L and ER showed additive effects on cell growth and their dual inhibition is suggested as a means to enhance the current effects of endocrine therapy [2]. Together, these two studies point to the potential value of targeting ER-coregulators.

There is increasing recognition that estrogen signaling may be important in other cancer types.

Furthermore, the major isoforms—ERα and estrogen receptor beta (ERβ)—have been shown to possess different functionalities. Di Zazzo et al. review data supporting a role for estrogen signaling in the epithelial to mesenchymal transition (EMT) in prostate cancer [3]. In this disease context, ERα is generally considered to mediate the adverse effects of estrogen including cell proliferation and survival while ERβ mediates inhibitory effects [3]. However, there is increasing recognition that the activity of ERβ is dependent on the balance of its splice variants present. ERβ1 represents the full-length form of ERβ and is the fully functional form. ERβ2 and ERβ5 are truncated forms of ERβ and are also found in prostate tissue and cancer cells; however, they cannot form homodimers or recruit regulators as ERβ1 can. Their expression is associated with disease progression and their effects appear to counteract those of ERβ1 [3]. The balance of expression levels and the formation of differing heterodimers are likely to lead to different outcomes within individual cancers [3].

Parallel findings for these isoforms and splice variants are also observed in ovarian cancer [4]. While ERα is associated with the promotion of growth and migration, ERβ has a suppressor role. Again, this inhibitory role of ERβ appears to be mediated by wild-type ERβ1 but can be neutralised by the opposing effects of ERβ2 and ERβ5 [4]. A role for the nongenomic membrane-bound G-protein coupled receptor (GPER1) is under investigation in this disease and evidence to support both a tumor-promoting and tumor-suppressive role has been obtained. Both anti-estrogens (tamoxifen and fulvestrant) and aromatase inhibitors (letrozole and anastrazole) have demonstrated antitumor activity in subgroups of ERα-high expressing cancers [4].

In AML, these opposing actions of ERα and ERβ continue to be the dominant view with ERα activation increasing the effects of chemotherapy while ERβ inhibits both leukemogenesis and leukemia cell proliferation and this area is reviewed by Roma and Spagnuolo [5]. The ratio of ERα to ERβ expression is important. Experimental data suggest that targeting ERβ with specific agonists may be a useful therapeutic approach with phytoestrogens (which have greater affinity for and act predominantly via ERβ) that, in particular, have interesting preclinical activity [5].

In breast cancer, mutations in the ligand binding domain of the ERα gene, *ESR1*, are increasingly being recognised as a mechanism of resistance to endocrine therapy in luminal disease and this area is reviewed by De Santo et al. [6]. These mutations are rare in untreated cancers but are selected for after endocrine therapy; they lead to constitutive estrogen-independent activation and hence therapy with aromatase inhibition (resulting in estrogen deprivation) becomes ineffective. Higher doses of anti-estrogens may still have some efficacy in these tumors. These mutations can be detected in circulating tumor DNA which is proving a useful method to monitor changes in metastatic disease [6]. The theme of monitoring responses is the subject of the review by Ella Jones et al. who describe the use of breast imaging to assess neo-adjuvant responses in ER-positive breast cancer [7]. The use of multimodality technologies, e.g., magnetic resonance imaging (MRI) and positon emission tomography, are increasingly being considered in this context. Multiparametric techniques have been developed for breast MRI and include volumetric (functional tumour volume), enhancement (background parenchymal enhancement), and diffusion (apparent diffusion coefficient) markers to assess response to therapy [7]. These approaches are likely to have an increasing part to play in the detection, diagnosis and response prediction for breast cancer in the future [7].
